# A survey of ethnomedicinal plants used to treat cancer by traditional medicine practitioners in Zimbabwe

**DOI:** 10.1186/s12906-020-03046-8

**Published:** 2020-09-14

**Authors:** Patrick Rutendo Matowa, Mazuru Gundidza, Lovemore Gwanzura, Charles F. B. Nhachi

**Affiliations:** 1 Clinical Pharmacology Department, University of Zimbabwe, Mt Pleasant, Harare, Zimbabwe; 2 Pharmaceutical Technology Department, Harare Institute of Technology, Belvedere, Harare, Zimbabwe; 3 Medical Laboratory Sciences, University of Zimbabwe, Mt pleasant, Harare, Zimbabwe

**Keywords:** Cancer, Traditional medicine, Traditional medicine practitioners, Medicinal plants

## Abstract

**Background:**

Traditional medicine plays an important role in health care provision in the developing world. A number of cancer patients have been found to be using traditional medicine as primary therapy and/or as complementary medicine. Cancer is one of the leading causes of morbidity and mortality globally among the non-communicable diseases. The aim of this study was to identify the plants used by traditional medicine practitioners (TMPs) in Zimbabwe to treat cancer.

**Methods:**

A structured questionnaire was used to interview consenting registered TMPs on ethnomedicinal plants they use to treat cancer. A review of published literature on the cited plants was also carried out. The practitioners were asked about the plants that they use to treat cancer, the plant parts used, type of cancer treated, other medicinal uses of the plants and preparation and administration of the plant parts.

**Results:**

Twenty (20) TMPs took part in the study. A total of 18 medicinal plant species were cited. The commonly treated types of cancer were breast, prostate, colon, skin and blood cancers with most plants being used to treat skin, blood and breast cancers, respectively. Of the medicinal plants cited, 44.4% were used to treat all cancer types. The most used plant parts were the roots (72.2%) and leaves (72.2%) followed by the bark (38.9%). The medicinal plants were used for multiple ailments. The most common plant preparation methods were infusion (72.2%) and decoction (66.7%) and the oral route of administration, as extracts and powder put in tea and porridge, was the most used.

**Conclusion:**

The frequently used plant parts were leaves and roots. The traditional uses of the medicinal plants cited in this study resonate well with their reported uses from other ethnopharmacological studies done in other parts of the world. The plants used by TMPs to treat cancer in Zimbabwe, if adequately explored, can be instrumental in the discovery and development of cancer drugs.

## Background

Traditional medicine refers to ways of protecting and restoring health that existed before the arrival of modern medicine [1]. The World Health Organisation (WHO) defines traditional medicine as native health practices, approaches, knowledge and beliefs that maybe applied either singular or in combination to treat, diagnose and maintain wellbeing [2]. It plays an important role in health care provision in the developing world and its use is also now significant in developed countries thus increasing commercial value. Traditional herbal medicines are naturally occurring plant and animal based substances with minimal or no industrial processing that are used to treat diseases within some healing practices [3]. Some people rely on these medicines to meet their health requirements. According to WHO, a third of the world’s population has no regular access to essential modern medicine [4]. In parts of Africa, Asia and Latin America, it is estimated that about half of the population faces shortages of minimum healthcare; due mainly to inadequacies in healthcare financing by the governments. This poses for glaring inequities in healthcare delivery in developing countries. Heavy burden of communicable diseases (HIV/AIDS, malaria, TB, pneumonia, diarrhoeas) coupled with the advent of growing threat of non-communicable diseases (NCDs) such as diabetes, cancer, hypertension, ischemic heart diseases; amongst many others, torment lives in developing countries [5]. The use of ethnomedicinal plants contribute to primary healthcare of people of the area that they are found [6] and thus contribute a rich health to human beings. About 80% of people living in rural settings in developing countries use traditional medicine for their primary health care needs [7, 8] and, generally, a number of cancer patients have been found to be using traditional medicine as primary therapy and/or as complementary medicine [9, 10].

A number of anticancer agents of plant origin, such as taxol, vincristine, vinblastine, etoposide, irinotecan and topotecan, are being used in clinical practice. Researches on ethnomedicine used to treat cancer continue to be pursued world over and the National Cancer Institute (NCI) is playing a huge role in research on medicinal plants used to treat cancer. The NCI has collected about 35,000 plant samples from 20 countries and screened, approximately, 114,000 extracts for anticancer activity [11]. All these efforts are because plant medicine plays a pivotal role in drug discovery and development. Also, these researches are meant to substantiate the medicinal claims and, on the other hand, help come up with well packaged finished drug products with well-defined dosage regimens for patients.

Cancer is the second leading cause of morbidity and mortality in the world amongst the NCDs [12–15]. A number of cancer deaths have been projected to increase from 7 million in 2002 to 11.5 million in 2030 [14]. Cancer is associated with the deterioration of quality of life, not only of the patients themselves, but also of spouse caregivers. Its diagnosis, length of hospitalisation, caregiving intensity and duration has a significant impact in determining the quality of life of spouse caregivers [16]. Treatment of the disease with some agents has been associated with deterioration of quality of life as well as induction of fatigue and some peripheral neuropathy; among a number of significant adverse effects. Common anti-cancer medicines frequently cause induction of a chemotherapy-induced peripheral neuropathy (CIPN) in which both large and small primary afferent sensory neurons are injured [17], while others are known to suppress the bone marrow; making the patients prone to infections and other diseases. Thus, development of medicines which offer optimal therapy of a condition and not have a negative impact on the quality of life of patients is of great importance. Many patients tend to complement or, in some instances, shun their conventional therapy with/for traditional medicine in an effort to minimise adverse effects.

A large proportion of the population in many developing countries relies on traditional practitioners and use of medicinal plants in order to meet healthcare needs. This is due to easy accessibility, efficacy on treatment and affordability in getting health services [18]. These traditional medicines have become more widely available commercially, especially in developed countries. In some countries, the production of these medicines is subjected to rigorous manufacturing standards [19]. Information about traditional medicine has been passed on from generation to generation mainly orally without substantive documentation. Some information could have been lost due to lack of documentation. Reliance on wild medicinal plants for primary healthcare by most communities has contributed to the continued preservation of knowledge of medicinal plants [20]. Documentation of the indigenous knowledge systems (IKS) on traditional medicine may assist in preserving medicinal plants and also patenting the IKS. The recognition of IKS is crucial for economic and cultural empowerment of indigenous people in particular, and the world at large. Incorporating indigenous knowledge into relevant policies such as health or climate policies can lead to the development of effective adaptation strategies that could be cost-effective, participatory and sustainable [21].

Despite the growing wide use of traditional medicine in Zimbabwe, the indigenous medicine knowledge on medicinal plants used to treat cancer is not widely documented. This prompted this particular ethnobotanical survey in an effort to address the gap. Documentation ensures that the traditional medicine knowledge is preserved and consequently leads to medicinal plants conservation. It also makes it easy for other subsequent studies that may focus on testing for activity of the identified plants against the claims.

## Methods

### Study area and data collection

The ethnomedicinal explorative study was conducted in the ten provinces of Zimbabwe. Practitioners were identified from the Traditional Medicine Practitioners Council (TMPC,) which falls under the Ministry of Health and Child Care (MoHCC). Convenience sampling was employed where participants (practitioners) available and willing to participate in the study were recruited and orally interviewed using a questionnaire as the instrument. Traditional medicine practitioners (TMPs) were visited in their homes and all present were invited for interviews. Before inclusion, the purpose of the study was explained to the TMPs. The researcher emphasized the enormous value which the TMPs (traditional healers and herbalists) input and contribution could make to the study as well as adding value to the already existing indigenous knowledge on traditional medicine practice in Zimbabwe. Data on the herbal plants used to treat cancer by consenting TMPs were recorded during visits to the practitioners. They were asked for knowledge about plants that they use against cancer, types of cancer treated, the plant parts used, methods of preparation and other medicinal uses of the plants. A taxonomist with the National Herbarium assisted with scientific names of plants cited using local names by some practitioners. This study mainly focused on data on medicinal plants used by TMPs to treat cancer. No plant samples were collected.

Ethical approval for the study was sort and obtained from Joint Research Ethical Committee for the University of Zimbabwe College of health Sciences and the Parirenyatwa Group of Hospitals (JREC), Medical Research Council of Zimbabwe (MRCZ) and the Traditional Medicine Practice Council (TMPC) of Zimbabwe. The researchers complied with the Convention on International Trade in Endangered Species of Wild Fauna and Flora.

Use value of the medicinal plants was determined by the frequency index (FI).

$FI=\left(\frac{FC}{N}\right)\times 100$. Where, FC is the number of participants who mention a particular plant species and N is the total number of participants [22].

Review of published literature; in the form of scientific journals, books and reports from international organisations; on the medicinal plants used by practitioners in the ethnomedicinal survey, was carried out.

## Results

### Demographics of the TMPs

As shown in Table 1, a total of 20 certified practitioners, 15 traditional healers, 4 herbalists and 1 naturopathologist, participated in the study. All fell under MoHCC’s TMPC. There were more male (75%) than female (25%) practitioners who participated in the study and confessed knowledge of plants that can be used to treat cancer. Most practitioners were above 50 years of age (mean = 64 ± 7.87) and a few were in the age range of 30–50 years (mean = 44 ± 7.01). None who could be below 30 years of age took part in the study. On educational level of the participants; the highest number (8 in 20) had attained secondary level, followed by primary and tertiary level practitioners; both categories had a frequency of 6 in 20 each. The participants belonged to varying religious groups; ranging from traditional, Moslem to Christianity. Among the participants, 60 and 10% claimed to be Christians and Moslems, respectively; whilst 30% claimed to follow the traditional way of worship. In terms of practice experience, 8 in every 10 practitioners had more than 20 years of experience in herbal medicine practice and all the practitioners interviewed had at least 5 years’ experience in their trade.

**Table 1:** Demographic details of respondents (*n* = 20)

Description	Frequency
Gender	
Male	15
Female	5
Education	
Primary	6
Secondary	8
Tertiary	6
Age	
< 30 years	0
30–50 years	6
> 50 years	14
Religion	
Christian	12
Muslim	2
Traditional	6
Practitioner qualification	
Traditional Healer	15
Herbalist	4
Naturopathologist	1
Experience of practitioner	
‹ a year	0
1–5 years	0
5–10 years	1
10–20 years	3
› 20 years	16

### Perceptions or understanding of characteristics of cancer

Most of the practitioners who participated in the study claimed to know and understand the disease, cancer. However, some relied on diagnosis or confirmation from doctors or hospitals. Most patients who visit the practitioners would have been diagnosed and tried conventional treatment before their visits. .

In describing what leads them to cancer diagnosis in patients, 8 practitioners highlighted chronic deep wounds which tend to widen over time. Other symptoms such as severe chronic pain of no specific origin, which occurs concurrently with tissue wasting and general loss of appetite, were given by 7 TMPs as indicators for cancer. Most TMPs associated painful breast lumps with breast cancer. Sixteen participants claimed to treat all forms of cancer. A few of the participants claimed that they were always successful in their management of any form of the disease, cancer. However, a substantial number alluded to the fact that sometimes their medicines may fail to yield the desired results and would refer the patients to conventional hospitals or superior practitioners.

The most common types of cancer encountered by the practitioners in their years of practice were breast, prostate, colon, skin cancer and cancer of the blood.

### Medicinal plants cited: Medicinal plants cited in this study are shown in Table 2.

#### Cited plants’ literature-reported medicinal uses or pharmacological activities: The medicinal uses or pharmacological activities, reported in literature, of the cited plants are indicated in Table 3.

##### Plants’ use value

The use value of the cited plants is given in Table 4. The plant species with more citations, indicated by relatively higher frequency indexes (FI), were said to have better use value than the ones with fewer citations or less FI. As such, *Cannabis sativa* L.*, Steganotaenia araliacea* Hochst., *Heteromorpha trifoliata* (Wendl.)Eckl. & Zeyh., *Kigelia africana* (Lam.) Benth.., *Bulbinella floribunda* (Thunb.) T.Durand. & Schinz., *Solanum incanum* L., *Pseudolachnostylis maprounelifolia* Pax [Agg]. and *Burkea africana* Hook., with more than one citation, had more use-value than the others in this study.

**Table 2:** Medicinal plants cited

Province	Plant Scientific Name	Family Name	Plant vernacular name	Cancers treated	Plant part(s) used	Other uses
Harare	*Steganotaenia araliacea* Hochst	Apiaceae	Musvodzambudzi	Breast, skin and blood	Bark, roots	Period pain, hypertension
	*Cannabis sativa* L.	Cannabaceae	Mbanje	All cancer types	Leaves, whole plant	Nausea, pain
Mashonaland	*Kigelia africana* Lam.	Bignoniaceae	Mubveve, Musonya	Blood, skin cancers	Bark, fruit, root, seeds	Malnutrition, diabetes,
Central			Muvhumati			antihelmintic
	*Asparagus africanus* Lam.	Asparagaceae	Rukato	Skin, prostate	Roots, leaves	Malaria, wounds
	*Cannabis sativa* L.	Cannabaceae	Mbanje	All cancers	Whole plant	Pain, chasing of evil spirits
Mashonaland	*Ricinus communis* L.	Euphorbiaceae	Mupfuta	Colon, blood	Roots, leaves, bark	Chest pain, stomach-ache,
East						constipation
	*Steganotaenia araliacea* Hochst	Apiaceae	Musvodzambudzi	All cancers	Bark, young leaves	STIs, infections
	*Bulbinella floribunda* (Thunb.)	Asphodelaceae	Chidzinganyoka	Breast, prostate, colon	Roots	Infections
	T. Durand &Schinz.					
	*Heteromorpha trifoliata* (Wendl.)	Apiaceae	Mhingano, imfenkulu	Skin, blood	Leaves, bark, roots	Aphrodisiac, asthma, infertility,
	Eckl. & Zeyh.					chest & back pain, anthelmintic fungal infections
Mashonaland	S*teganotaenia araliacea* Hochst.	Apiaceae	Musvodzambudzi	Skin cancer	Stem bark	Eye sores, STIs
West	*Duranta erecta* L.	Verbenaceae		All cancer types	Leaves, roots, bark, Fruits	Pain, infections (fungal & bacterial)
Masvingo	*Solanum incanum* L.	Solanaceae	Nhundurwa	Skin, breast & blood cancers.	Fruits, roots, leaves	Sore eyes, snake bites, ulcers, toothache.
	*Moringa oleifera* Lam.	Moringaceae	Moringa	All cancer types	Leaves, roots, seeds	Malnutrition, diabetes, antimicrobial
Manicaland	*Solanum incanum* L.	Solanaceae	Nhundurwa	Skin and blood cancers	Fruits, leaves, roots	Wounds, snake bites
	*Ximenia caffra* Sond.	Ximeniaceae	Munhengeni, mutsvanzva, Umthunduluka	All cancer types	Roots, fruits, seeds	Antimicrobial, STIs, malnutrition, aphrodisiac
	*Kigelia africana* (Lam.) Benth.	Bignoniaceae	Mubveve	Breast, prostate & skin cancers.	Fruits, leaves, bark	Eczema, toothache, pain, diarrhoea, diabetes, skin infections.
	*Pseudolachnostylis maprouneifolia* Pax [Agg].	Phyllanthaceae	Mutsonzwa, mukuvazviyo	Eye and skin cancers	Leaves	Skin rashes and infections
Matebeleland	*Hypoxis hemerocallidea*	Hypoxidaceae		Blood cancers	Corms	Diabetes
North	(Fisch. & C.A.Mey.) *Heteromorpha trifoliata* (Wendl.)	Apiaceae	Mhingano, imfenkulu	Lung, prostate, breast,	Leaves, bark, roots	Infections, pain, immune system booster,
	Eckl. & Zeyh.			skin cancers		respiratory ailments.
	*Prunus persica* (L.)Batsch	Rosaceae	Mupichisi	Skin cancer	Seeds, stem bark	Antimicrobial
	*Cannabis sativa* L.	Cannabaceae	Imbanje, mbanje	All cancer types	Leaves, seeds	Endurance, analgesic
Matebeleland	*Burkea africana* Hook.	Fabaceae	Umnondo, mukarati	Blood, colon cancers	Roots	Infections, abdominal pain
South	*Lantana rugosa* (camara) Thunb.	Verbenaceae	Ubuhobe, mubanda	Blood, skin cancers	Leaves, roots	Asthma, chest pain, ulcers, skin itchiness, growth enhancement in children
Bulawayo	*Hibiscus sabdariffa* L.	Malvaceae		All cancer types	Calyces	Malnutrition, infections
	*Cannabis sativa* L.	Cannabaceae	Imbanje, mbanje	All cancer types	Leaves, seeds	Vomiting, pain, epilepsy
	*Bulbinella floribunda* (Thunb.) T. Durand &Schinz.	Asphodelaceae	Chidzinganyoka	Colon, skin, breast cancers.	Leaves	Snake repellent
	*Heteromorpha trifoliata* (Wendl.) Eckl. & Zeyh.	Apiaceae	Mhingano, imfenkulu	Breast, blood cancers	Roots, leaves	Antimicrobial
Midlands	*Sutherlandia frutescens*(L.)R.Br.	Fabaceae		All cancer types	Shoots	Diabetes, fever, analgesia, colds & flu, haemorrhoids
	*Burkea africana* Hook.	Fabaceae	Mukarati, umnondo	Blood, prostate cancers	Roots, stem bark	Immune system booster, anti- inflammatory
	*Pseudolachnostylis* *maprouneifolia* Pax [Agg].	Phyllanthaceae	Mutsonzwa, mukurazviyo	All cancer types	Leaves	Antimicrobial

**Table 3:** Cited plants’ literature-reported medicinal uses or pharmacological activities

Scientific Name	Family Name Name	Vernacular	Plant part utilised	Medicinal Uses (from literature)
*Steganotaenia araliacea* Hochst.	Apiaceae	Musvodzambudzi	Stem and root bark	Diarrhoea, infections, antimitotic activity [23, 24].
*Cannabis sativa* L.	Cannabaceae	Mbanje	Whole plant	Asthma, nausea, emesis, insomnia, anxiety, anorexia, malaria, epilepsy, psychosis, psoriasis [25–28].
*Kigelia africana* (Lam.) Benth.	Bignoniaceae	Mubveve	Fruit, leaves, bark, roots	Pneumonia, toothache, dysentery, ulcers, antifungal, psoriasis, cancer, eczema, antibacterial, malaria, analgesic, diabetes, aphrodisiac [29–31].
*Pseudolachnostylis maprouneifolia* Pax [Agg].	Phyllanthaceae	Mutsonzwa/Mukurazviyo	Leaves	Eye cancer [32].
*Heteromorpha trifoliata* (Wendl.) Eckl.&Zeyh.	Apiaceae	Mhingano	Stem bark	Cancer [31].
*Burkea africana* Hook.	Fabaceae	Mukarati	Stem bark	Antioxidant [33, 34].
*Moringa oliefera* Lam.	Moringaceae	Moringa	Whole plant	Cancer, diabetes, hypertension, high cholesterol, infections, immune system booster [35–37].
*Ximenia caffra* Sond.	Ximeniaceae	Nhengeni	Fruit, leaves, root, seed	Diarrhoea, bilharzia, malaria, infections, intestinal worms, STIs, infertility, pain [38].
*Ricinus communis* L.	Euphorbiaceae	Mupfuta	Bark, leaves, seeds, oil	Pain, diabetes, tumours, asthma [39, 40].
*Hypoxis hemerocallidea* Fisch.&C.A.Mey.	Hypoxidaceae		Corms	Cancer, nervous disorders, UTIs, HIV/AIDS, diabetes, hypertension, arthritis, colds and flu [41–43].
*Prunus persica* (L.)Batsch	Rosaceae	Muphichisi	Bark, leaves	Common cold, diarrhoea, haemorrhoids [44, 45].
*Duranta erecta* L.	Verbenaceae		Leaves	Headache, toothache, wound healing, diuretic, malaria [46, 47].
*Bulbinella floribunda* (Thunb.)T.Durand&Schinz.	Asphodelaceae	Chidzinganyoka	Roots	Infections, bleeding, acne, cold sores, chapped lips, cracked heels, eczema [48].
*Sutherlandia frutescens* (L.)R.BR.	Fabaceae		Shoots	Cancer, HIV/AIDS, diabetes, influenza, depression, arthritis, peptic ulcers, gastritis, reflux oesophagitis, chronic fatigue syndrome [43, 49–51].
*Lantana rugosa* Thunb.	Verbenaceae		Roots, leaves	Ulcers, malaria, wound infections, tumors, eczema, pain [52, 53].
*Solanum incanum* L.	Solanaceae	Nhundurwa	Leaves, fruit, roots	Sore throat, stomach ache, snake bites, ringworm, warts, fever, liver disorders, menstrual disorders [54, 55].
*Hibiscus subdariffa* L.	Malvaceae		Leaves	Cancers, hypertension, hyperglycemia, high cholesterol [56–59].
*Asparagus africanus* Lam.	Asparagaceae	Rukato	Roots	Analgesic, malaria, haemorrhoids, STIs, diuretic, aid in childbirth [31, 60].

**Table 4:** Use value of the cited medicinal plants

Plant name	Frequency	FI
*Asparagus africanus*	1	0.05
*Bulbinella floribunda*	2	0.10
*Burkea africana*	2	0.10
*Cannabis sativa*	4	0.20
*Duranta erecta*	1	0.05
*Heteromorpha trifoliata*	3	0.15
*Hibiscus subdariffa*	1	0.05
*Hypoxis hemerocallidea*	1	0.05
*Kigelia africana*	2	0.10
*Lantana rugosa*	1	0.05
*Moringa oleifera*	1	0.05
*Prunus persica*	1	0.05
*Pseudolachnostylis maprouneifolia*	2	0.10
*Ricinus communis*	1	0.05
*Solanum incanum*	2	0.10
*Steganotaenia araliacea*	3	0.15
*Sutherlandia frutescens*	1	0.05
*Ximenia caffra*	1	0.05

##### Medicinal plant parts used

The different plant parts used by TMPs to make their medicinal products for patient administration are shown in Table 5. These plant parts include leaves, roots, bark, seeds, the fruit and, in one case, the whole plant. The leaves and roots were the most used plant parts among the plants cited. In 13 plant species (72.2%), roots and leaves were the useful plants parts, in 7 plant species (38.9%), the bark was used; whilst seeds and fruits were the plant parts used in 5 (27.8%) and 4 (22.2%) plant species. It was also noted that, as shown in Table 3, these plant species were not only useful for treatment of cancer, but also, for multiple other medical ailments. A single plant could be used for more than one ailment. It was also important to note that, out of the plant species obtained in this study, a number of them were cultivated or brought from neighbouring countries such as South Africa and Botswana by some of the practitioners, namely herbalists. These plants could no longer be easily found in the wild, locally. These species included *Sutherlandia frutescens* (L.)R.Br.*, Prunus persica* (L.) Batsch*, Duranta erecta* L.*, Bulbinella floribunda* (Thunb.) T. Durand & Schinz*, Cannabis sativa* L. and *Hibiscus sabdariffa* L.

**Table 5:** Parts of medicinal plants used to treat cancer

Plant parts used	Number of plant species	Percentage
Bark	7	38.9
Roots	13	72.2
Leaves	13	72.2
Whole plant	1	5.6
Fruits	4	22.2
Seeds	5	27.8

##### Types of cancer treated

Table 6 shows the different plant species that were used by the TMPs in this study, to treat different types of cancer. Eight [8] plant species (44.4%) were used to treat skin cancer, 38.9% (7 plant species) were used for cancer of the blood, 33.3% (6 plant species) were used to treat breast cancer, 27.8% (5 plant species) were used for prostate cancer, 16.7% (3 plant species) were used to treat cancer of the colon and a single plant species (5.65%) was useful in treating cancers of the eye and lung cancer in each case. Of all the cited plants species, 44.4% were used to treat all types of cancer.

**Table 6:** Types of cancer treated by the different traditional medicinal plants

Type of cancer	Plants used	Percentage
Skin	*S.araliacea, K.africana, H.trifoliata, S.incanum,* *P.maprouneifolia, P.persica, L.rugosa, B.floribunda*	44.4
All cancers *H.sabdariffa,*	*C.sativa, P.maprouneifolia, S.frutescens,* *S.araliacea, D.erecta, M.oleifera, X.caffra*	44.4
Blood	*S.araliacea, H.hemerocallidea, B.africana, L.rugosa,* *H.trifoliata, K.africana, S.incanum*	38.9
Breast	*S. araliacea, B.floribunda, S.incanum, K.africana,* *H.trifoliata, P.maprouneifolia*	33.3
Prostate	*H.trifoliata, B.africana, A.africanus, B.floribunda,* *K.africana*	27.8
Colon	*R.communis, B.floribunda, B.africana*	16.7
Eye	*P.maprouneifolia*	5.6
Lung	*H.trifoliata*	5.6

##### Cited plants’ material preparation/administration for medicinal purposes

As shown in Table 7, the medicinal plants were largely administered to patients as extracts following decoction (66.7%) or infusion (72.2%). The most reported administration route was oral as a decoction, infusion, by chewing or put in tea or porridge. In some instances, plant extracts could be applied as a wash without using a drying towel; and also as fresh leaf sap or fruit macerate onto the affected body area. Tinctures (11.1%) were also prepared and *Cannabis sativa* L., in addition to its other administration routes, could also be smoked for therapeutic purposes.

**Table 7:** Plant material preparation/administration for medicinal purposes

Scientific name	Plant part utilised	Preparation
*Asparagus africanus*	roots, leaves	decoction, infusion
*Bulbinella floribunda*	roots, leaves	infusion, leaf sap applied topically.
*Burkea africana*	stem bark, roots	decoction
*Cannabis sativa*	whole plant	infusion, decoction, smoked, chewed, put in tea.
*Duranta erecta*	leaves, roots, fruits, bark	pulverised powder put in water and supernatant extract orally administered.
*Heteromorpha trifoliata*	leaves, bark roots	infusion, decoction
*Hibiscus sabdariffa*	calyces, leaves	fresh leaves chewed, infusion
*Hypoxis hemerocallidea*	corms	powder put in porridge, infusion.
*Kigelia africana*	fruits, roots, leaves, bark	decoction, infusion, extract applied onto the body as a wash, powder put in porridge.
*Lantana rugosa*	roots, leaves	decoction.
*Moringa oleifera*	roots, leaves, seeds	infusion, decoction, applied onto affected area, powder put in porridge
*Prunus persica*	stem bark, leaves, seeds	decoction, infusion
*Pseudolachnostylis maprouneifolia*	leaves	infusion instilled into affectedeyes, decoction
*Ricinus communis*	leaves, roots, bark	decoction
*Solanum incanum*	leaves, fruits, roots	fruit macerate applied onto affected area, infusion, tincture
*Steganotaenia araliacea*	stem bark, roots, leaves	decoction, infusion, tincture
*Sutherlandia frutescens*	shoots	infusion
*Ximenia caffra*	fruits, roots, seeds	infusion, decoction, chewed.

## Discussion

There are a number of traditional practitioners (traditional healers, naturopathologists and herbalists) herein collectively termed TMPs, dotted around the country in different provinces. However, getting to these practitioners or getting information from these practitioners was a huge challenge. Nonetheless, some understood the importance of the study and volunteered their knowledge on the subject matter.

The demographics of the study participants are a shown in Table 1. In this study, more male [15] than female [5] practitioners participated.

Initially, the ratio of female to male practitioners who were notified of the study was 1:1,5. However, in the final participants group the ratio came to 1:3 (female to male practitioners). The explanation behind this observation could be that more female than male practitioners were reluctant to give out the information sort for and thus could not take part in the study. Nonetheless, the figures show that the TMPs who treat cancer in Zimbabwe are predominantly males. TMPs who volunteered in the study were from different religion backgrounds, as shown in Table 1. Most of the participants [12] had a Christianity background, followed by traditional religion [6] and lastly, Muslim [2]. This observation dovetails with the general religion distribution in the country where 84% of the population is reported to be Christians [61]. As such, the participant sample reflects a mimicking of TMPs distribution across the religion divide in the country. It also shows that people could still practice traditional medicine regardless of their religion background.

Although all the practitioners claimed to know and understand cancer as a disease, they had varying descriptions of the condition thus its diagnosis varied considerably amongst the practitioners. However, they all concurred to the fact that most patients would consult the TMPs late after being diagnosed of the disease and commenced on conventional treatment. The TMPs highlighted this delay by patients to consult their expertise as one of the contributory factor in cases where their treatments may be deemed to have failed.

Table 2 illustrates the different plants cited by practitioners to be useful in treatment of cancer. Among the most cited plants was *Cannabis sativa* L. which was used in cancer mainly for its analgesic, anti-nausea and antiemetic properties. Moreover, some claimed it was also helpful in reducing the spread of the disease. The findings dovetail with reports on the medical uses of cannabis elsewhere (Table 3) wherein it is reported that cannabis was found to be useful as an analgesic as well as for chemotherapy-induced nausea and vomiting [25], [62]. The Arab physicians were reported to be users of *Cannabis sativa* L. extract for its diuretic, anti-emetic, anti-epileptic, anti-inflammatory, analgesic and antipyretic properties. They would also apply the seed oil on to hardened tumours and the tumours would dissolve [26]. There is also evidence of cannabis efficacy in the treatment of insomnia, anxiety and asthma among other uses. Cannabidol, a cannabinoid phytoconstituent, was thought to be responsible for the antipsychotic activity of cannabis [27]. The other use of cannabis in cancer is related to its ability to increase appetite, a phenomenon very useful in cancer patients where appetite is normally suppressed. Scientific studies, though inconclusive, had hinted on the potential efficacy of cannabis as an anti-tumour agent and needed more exploration [62]. Studies on the cannabis plant extract phytoconstituents indicate the presence of over 100 cannabinoids, 9-THC being the most potent and responsible for the psychoactive effects [63]; terpenoids, flavonoids and alkaloids [64]. Given the high frequency index of cannabis in this study, it is prudent to pursue its efficacy in cell growth inhibition or as a cytotoxic agent.

The other most cited plants across the country were *Steganotaenia araliacea* Hochst., *Heteromorpha trifoliata* (Wendl.)Eckl. & Zeyh., *Kigelia africana* (Lam.) Benth., *Bulbinella floribunda* (Thunb.) T. Durand & Schinz., *Solanum incanum* L., *Pseudolachnostylis maprouneifolia* Pax [Agg]. and *Burkea africana* Hook. as indicated in Table 4. This implies that these medicinal plant species have better use-value than the other plants cited only once in this study. The list of plants obtained and their traditional uses can be compared with what came out of other studies that concentrated on the cytotoxicity of certain medicinal plant extracts. For example, *Kigelia africana* (Lam.) Benth.***,*** highlighted by some practitioners in this study’ has been found from other studies to be useful for skin ailments (eczema, psoriasis, fungal infection, boils), diarrhoea, haemorrhoids, fever, malaria, diabetes as well as having anti-cancer properties [29]. The bioactive constituents found to be present in all the plant parts include iridoids, naphthoquinones, flavonoids, terpenes and pheneylethanoglycosides [29, 65]. The stem bark and fruit extracts were found to be cytotoxic against cancer cell lines [29]. Another study revealed anticancer activity of the fruit extract against cervical and colorectal cancer cell lines [30]. *Hibiscus sabdariffa* L. and *Pseudolachnostylis maprouneifolia* Pax [Agg]. have been reported for their use as cytotoxic agents, among other activities [32, 56]. The high polyphenol content in dried leaf extract of *Hibiscus sabdariffa* L. have been linked to its activity against leukaemia and gastric carcinoma [57]. Studies have also shown *Hibiscus sabdariffa* L. leaf extract-induced apoptosis in human prostate cancer cells [58]. Other medicinal plants that have been reported for their actual or potential anti-cancer activities include *Hypoxis hemerocallidea* Fisch.&C.A.Mey [66], [67]., *Sutherlandia frutescens* (Lam.)R.BR [50]. as well as *Solanum incanum* L. [54]. *Hypoxis hemerocallidea* Fisch.&C.A.Mey.*,* also referred to as the miracle plant [68], apart from other documented activities such as hypoglycaemic and antioxidant properties [41]; has been shown to have activity against cancerous and premalignant cancer cells [42]. The herbal extract of *Solanum incanum* L. has been found to possess anticancer, antinociceptive, antipyretic, antispasmolytic, oretic, hypoglycemic and antimicrobial activities [54]. Notable active components thought to be responsible for the anticancer activity are glycoalkaloids; which include solamargine, solasodine and solasomine. Solarmagine was found to act by inducing cancer cells apoptosis [55]. Cancer bush, *Sutherlandia frutescens* (L.)R.Br*,* is native to South Africa and has been traditionally used in the treatment and prophylaxis of cancer [43, 49]. An in-vitro study demonstrated a concentration-dependent inhibition of several tumour cell lines as well as antioxidant activity in reducing free radical cations [49]. *Lantana rugosa* (camara) Thunb. root and leaf extracts were shown to have activity against cancer cell lines [52] and the plant has been used in folklore medicine for its antipyretic, antimalarial and antimutagenic antitumor, analgesic, antimicrobial and anti-eczema properties [52, 53]. *Prunus persica* (L.) Batsch. roots, bark, fruits, leaves and flowers found uses in treating haemorrhoids, diarrhoea and the common cold [44]. Its glycosides bioactive constituents were reported to have activity in the inhibition of tumour growth as well as chemoprevention [45]. Other plants highlighted by traditional medicine practitioners in the study that also had anticancer uses or activities reported in elsewhere include *Moringa oleifera* Lam., *Heteromorpha trifoliata* (Wendl)Eckl. & Zeyh., *Ximenia caffra* Sond., *Ricinus communis* L., *Steganotaenia araliacea* Hochst.*,* as shown in Table 3. Some plants were not reported for their anticancer activities in literature but were being used to treat cancer by practitioners in this study. Such plants include *Duranta erecta* L., *Asparagus africanus* Lam., *Bulbinella floribunda* (Thunb.) T. Durand & Schinz. and *Burkea africana* Hook. These plants’ pharmacological significance could be due to the presence of phytoconstituents such as flavonoids, saponins, phenolics, glycosides, alkaloids and tannins in the extracts; which have been found to confer activity against cancer cells. The findings from the present study, therefore, relate to some of the plants’ uses that have been reported by other researchers as demonstrated above. This could be an indication of a strong primary health care provision offered by these TMPs using medicinal plants.

Table 2 shows that the roots and the leaves were the most commonly used plant parts from this study. This is in-sync with other studies where it was also noted that leaves and roots are generally the most frequently used plant parts in traditional medicine [69, 70]. From Table 4, the most treated cancers by the practitioners in this study were cancers of the skin, blood and breast. This trend could be explained from different angles. It could be because blood and skin cancers are also related to other conditions such as the infectious disease, HIV/AIDS, which has a significant prevalence in Zimbabwe. Breast cancer is also one of the most common types of cancer globally and thus very possible for the practitioners to encounter a number of patients suffering from it. Surprisingly, there was no mention of cervical cancer in the study, whose incidence and prevalence has been a cause of concern in the health fraternity in Zimbabwe.

The plants’ material preparation included infusion, decoction, pulverised powder, tinctures and burning for inhalation or smoking. In most cases the plants’ parts would be administered orally as extracts or as pulverised powder in porridge or tea. The practitioners were not clear on the doses administered as well as frequency and duration of treatment. However, generally, the commonly alluded to measure of dose was one to two tablespoonful of either the powder or extract administered twice or three times a day until the condition improves. In cases where patients fail to improve, combination of the plants’ extracts would be administered so as to get additive or synergistic effects. The medicinal plants were reported by participants to be very safe with insignificant adverse effects. Effects such as dizziness and diarrhoea were found to be common in many patients but reported as an indication that ‘the herbs were working’.

Generally, practitioners were not free to give out information. Some were not willing to divulge information about certain medicinal plants, especially the ones they felt the activities to be very powerful. Apparently, such plants serve as the reference trademark of patients from other practitioners and the community at large, hence making them gurus of their trade and earning them community respect as well. Some were adamant to keep this knowledge to themselves as something belonging to their own guarded family secrete and would only divulge to specific chosen family member who would carry own with the legacy. In some of the cases encountered, divulging and/or collection of such information would be subsequent to certain traditional procedures or consultations which would involve some rituals and fees. The major limitation to the study was to do with practitioners’ reluctance with information. Many thought that giving away their knowledge for free would disadvantage them as probably drugs would be discovered from that knowledge without them benefiting from the results. They would rather die with their knowledge or only pass it to their chosen family member who would also hold the information sacredly. Some would only offer their information at a price, for example, a herd of cattle.

Education of TMPC members on the importance of indigenous medicine systems can help researchers in getting important information from TMPs for research purposes much more easily. Also, assistance to the TMPs on issues concerning patents of indigenous knowledge on traditional medicine could go a long way in making sure that indigenous knowledge is preserved and can be accessed by those who may need it whenever they need it.

Unsustainable plant harvesting or collection was found to be rampant among the practitioners, especially where plant roots were of use, thereby posing as a serious threat to plants extinction. There is need for education on sustainable utilisation of plant resources which does not lead to deforestation and extinction of some of the valuable plant species.

A limitation of this study is the small sample size of TMPs who participated. A larger sample size with more participants could have resulted in more plant species being cited as well as broader data and knowledge about medicinal plants use in cancer treatment by TMPs. Follow-up studies with larger sample sizes may yield more indigenous knowledge about plant medicine use in cancer treatment.

## Conclusion

TMPs from different areas used different plants for the treatment of the same type of cancer or disease. However, in some cases; similar plants were used by practitioners from different areas. The medicinal plants were used to treat multiple medical conditions by different practitioners. Cancers of the skin, blood and breast were the most commonly treated cancers by practitioners in this study. Roots and leaves were the plant parts mostly used. Oral administration was the most common route of administration by the practitioners, however, other methods of administration such as sap or fruit macerate topical application, body wash and smoking were also utilised. The traditional uses of medicinal plants cited in this study resonate well with those from pharmacological studies on the same plants done in other parts of the world. The work revealed a number of plants used by TMPs to treat cancer which, if explored, may act as templates or leads for cancer drugs discovery and development. Moreover, toxicity studies on these plants need exploration as they may pose potential toxicity in some patients.

## Data Availability

All data generated or analysed during this study are included in this article. The materials and data of our study are available to other researchers upon request.

## Abbreviations

NCDs: Non-Communicable Diseases
CIPN: Chemotherapy-Induced Peripheral Neuropathy
WHO: World Health Organisation
NCI: National Cancer Institute
IKS: Indigenous Knowledge Systems
HIV/AIDS: Human Immunodeficiency Virus/Acquired Immunodeficiency Syndrome
TB: Tuberculosis
DNA: Deoxyribonucleic Acid
TMPC: Traditional Medicine Practitioners Council
MoHCC: Ministry of Health and Child Care
JREC: Joint Research Ethical Committee for the University of Zimbabwe College of Health Sciences and the Parirenyatwa Group of Hospitals
MRCZ: Medical Research Council of Zimbabwe
FI: Frequency Index
TMPs: Traditional Medicine Practioners
STIs: Sexually Transmitted Infections
